# Supplementary material for: Identification of positive and negative regulators of antiviral RNA interference in Arabidopsis thaliana
Source: Nat Commun. 2022 May 30;13:2994. doi: 10.1038/s41467-022-30771-0 (PMC9151786; doi:10.1038/s41467-022-30771-0)
Supplement: Supplementary file 1 — Supplementary Information [file 41467_2022_30771_MOESM1_ESM.pdf]

Identification of positive and negative regulators of antiviral RNA interference in *Arabidopsis thaliana*

Liu *et al.*

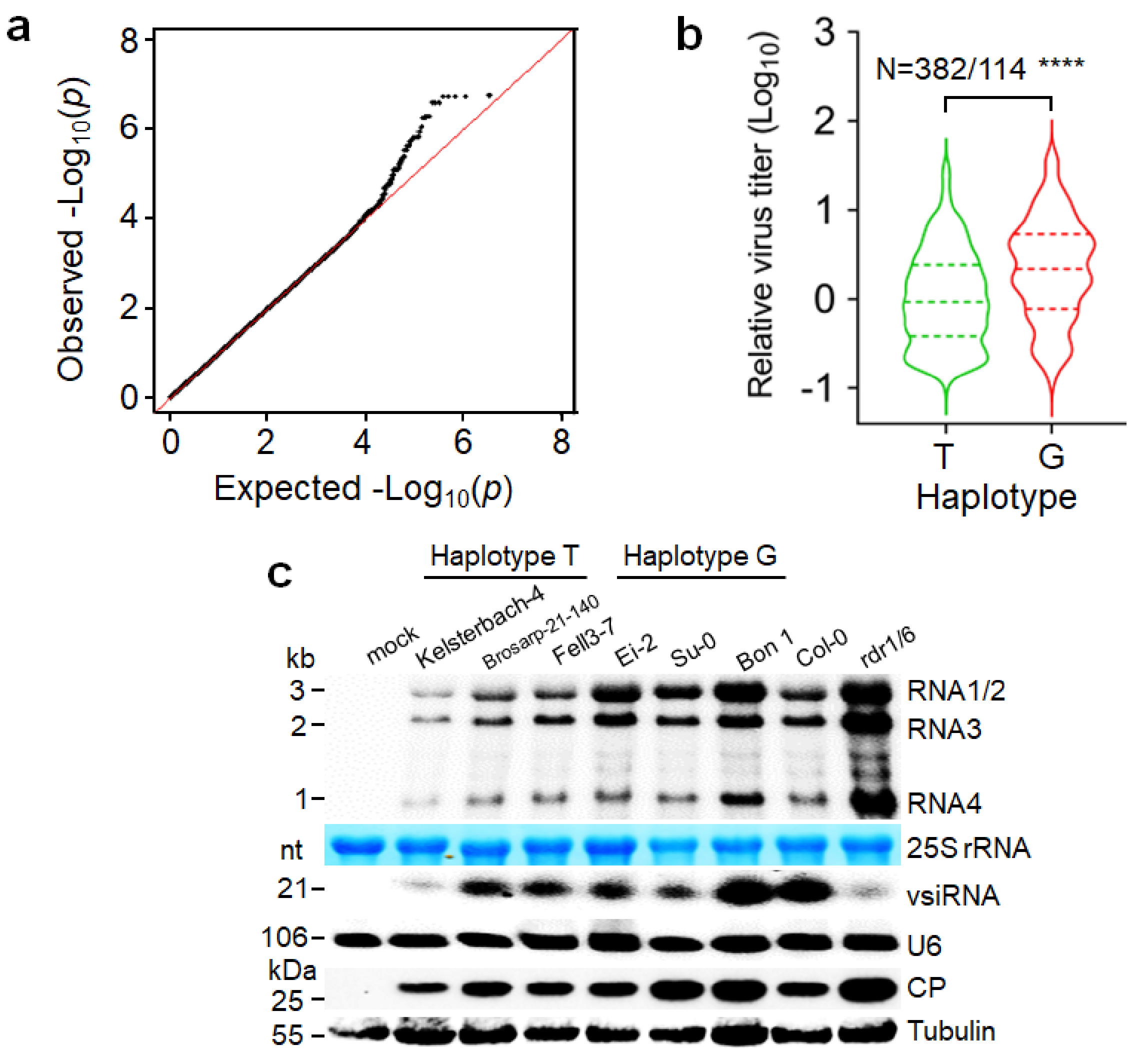

Supplementary Figure 1. GWAS analysis of the quantitative virus resistance phenotype. a, Quantile-quantile plot of p values calculated by GWAS. x axis, expected p values (log-transformed) of SNPs based on null distribution; y axis, observed p values (log-transformed) of SNPs. The data points that substantially deviate from the diagonal line (x = y) indicated the potential associations between SNPs and the quantitative virus resistance phenotype. **b**, Virus titer comparison of haplotypes based on the genotype of the most significantly associated SNP found in *RDO5*. The bars within violin plots represent 25th percentiles, medians, and 75th percentiles. N, the number of accessions from each haplotype. **** indicates significant difference (two-sided t test, p=2.65e^{-8}). See supplementary data 1 for data resource. **c**, Characterization of CMV-Δ2b infection in Col-0, *rdr1/6* and natural accessions from haplotypes as indicated. CMV-Δ2b replicated to lower levels in 4 selected accessions of haplotype T including Col-0 than the 3 accessions of haplotype G and the experiment was repeated three times independently with similar results. The source data underlying blots in (**c**) are provided as a Source Data file.

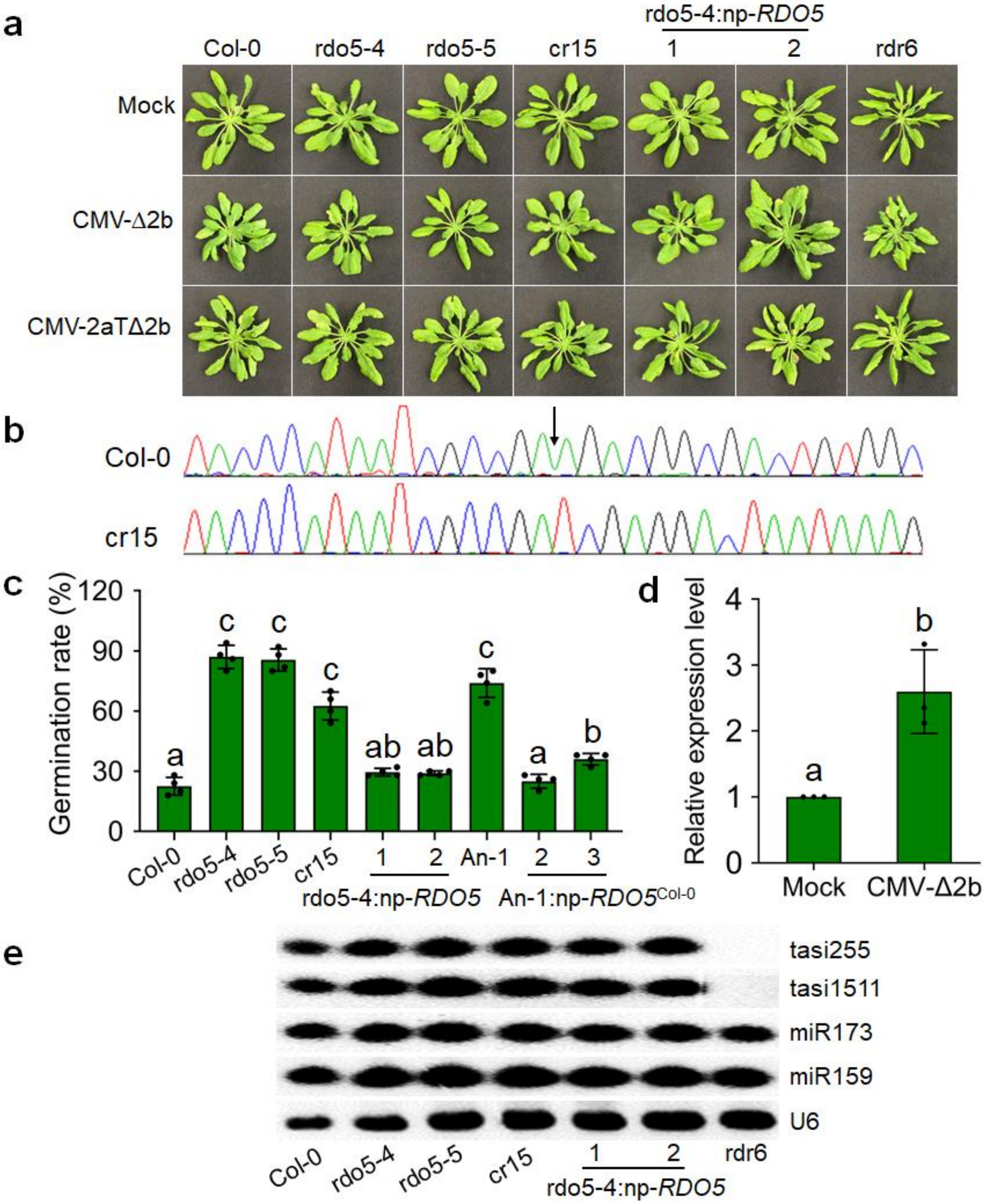

Supplementary Figure 2. Characterization of *rdo5* mutants. a, Wild-type and mutant plants were photographed 2 weeks post-inoculation with buffer (mock), CMV-Δ2b or CMV-2aTΔ2b. No obvious phenotypic differences were observed among Col-0, *rdo5* mutants and the complemented lines with or without infection in contrast to the *rdr6* mutant. **b**, DNA sequencing of Col-0 and the edited mutant *cr15*. Black arrow indicates the position of the introduced

deletion. **c**, Seed dormancy assay to determine the germination rates of Col-0, An-1, *rdo5* mutants and the *RDO5*-complemented lines. Data are means ± SEM from four replicates and letters indicate significant differences (one-way ANOVA, Duncan, $p<0.05$). **d**, RT-qPCR analysis of *RDO5* mRNA levels in Col-0 with or without CMV-Δ2b infection using EF1α (At5g60390) mRNA as internal control. Data are means ± SEM from three independent experiments and letters indicate significant differences (two-sided t test, $p=0.0121$). **e**, Northern blot detection of plant endogenous small RNAs (tasi255, tasi1511, miR173 and miR159) in healthy wild-type and mutant plants as well as the complemented lines as indicated, the experiment was repeated three times independently with similar results. Black dots in **c** and **d** represent the individual values. The source data underlying virus infection symptoms in (**a**), Germination rates in (**c**), qRT-PCR data in (**d**) and blots in (**e**) are provided as a Source Data file.

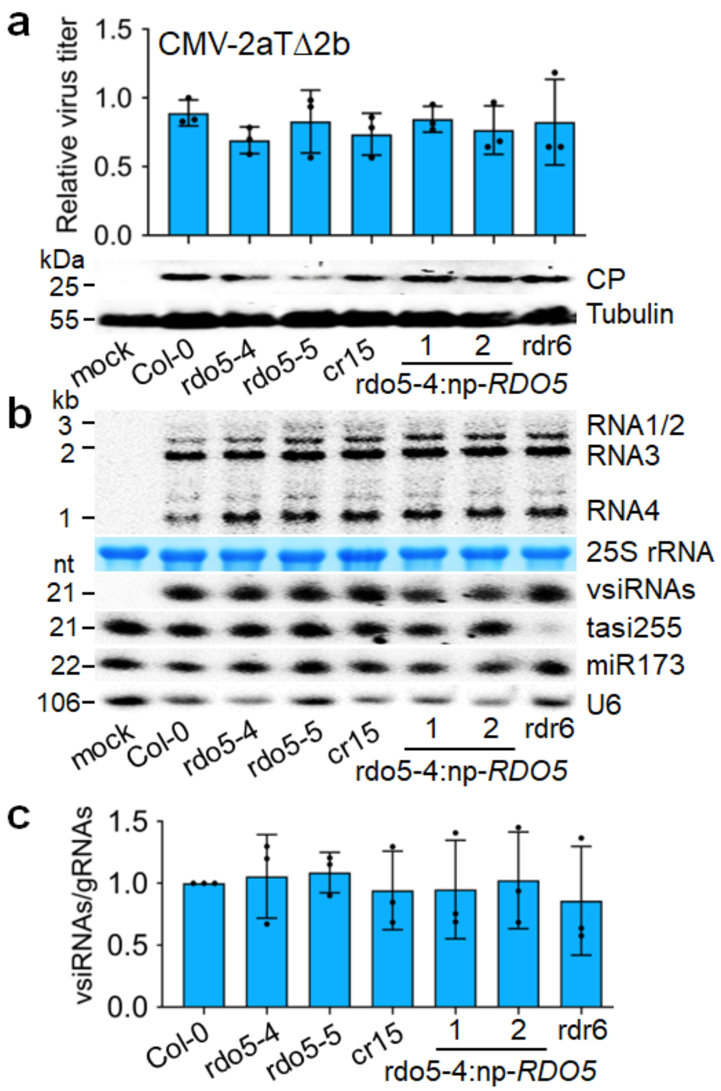

Supplementary Figure 3. Characterization of CMV-2aTΔ2b infection in *rdo5* mutants. a, ELISA and Western blot detection of the viral coat protein (CP) accumulation and **b**, Northern blot detection of the viral RNAs 1-4 and vsiRNAs, as well as plant endogenous tasi255, miR173 and U6 RNA in Col-0, *rdr6* mutant, *rdo5* mutants and the complemented lines at 2 weeks post-inoculation with CMV-2aTΔ2b. **c**, Ratios of vsiRNAs/gRNAs were calculated from Phosphor-imager readings of Northern hybridization signals in (**b**) and the ratio in Col-0 was set as 1. Data presented are means ± SEM from three replicates (**a**) or independent experiments (**c**) and no significant difference in either virus accumulation levels or vsiRNAs/gRNA ratios was found among wild-type and mutant plants examined (one-way ANOVA, Duncan, p>0.05), black dots represent the individual values. The source data underlying blots in (**a**) and (**b**), ELISA data in (**a**) and ratio data in (**c**) are provided as a Source Data file.

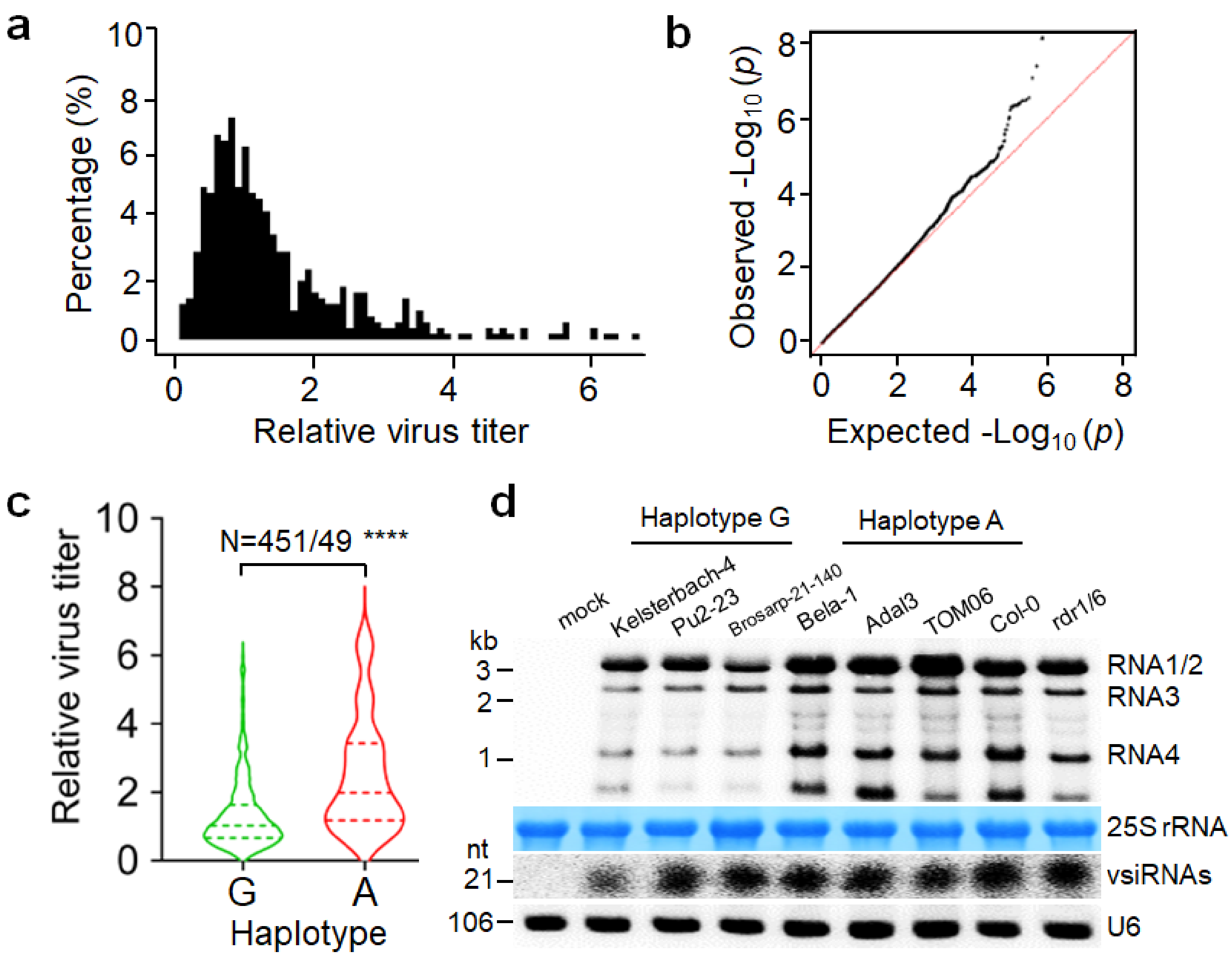

Supplementary Figure 4. Natural variation of the susceptibility to wild-type Q-CMV. a, Distribution of virus accumulation levels in 500 wild-collected *A. thaliana* accessions at 2 weeks post-infection as determined by RT-qPCR detection of the viral RNA3. **b**, Quantile-quantile plot of p values calculated by GWAS. x axis, expected p values of SNPs based on null distribution; y axis, observed p values of SNPs. The data points that substantially deviate from the diagonal line (x = y) indicated the potential associations between SNPs and the quantitative phenotype of virus accumulation levels. **c**, Virus titer comparison of haplotypes based on the genotype of the highest SNP found in *VIR1* screen. The bars within violin plots represent 25th percentiles, medians, and 75th percentiles. N indicates the number of accessions from each haplotype. **** indicates significant difference (two-sided t test, $p=6.21e^{-12}$). See supplementary data 1 for data resource. **d**, Characterization of Q-CMV infection in Col-0, *rdr1/6* and natural accessions from haplotypes as indicated. Q-CMV replicated to lower levels in 3 selected accessions of haplotype G than the 4 accessions of haplotype A including Col-0 and the experiment was repeated three times independently with similar results. The source data underlying blots in (**d**) are provided as a Source Data file.

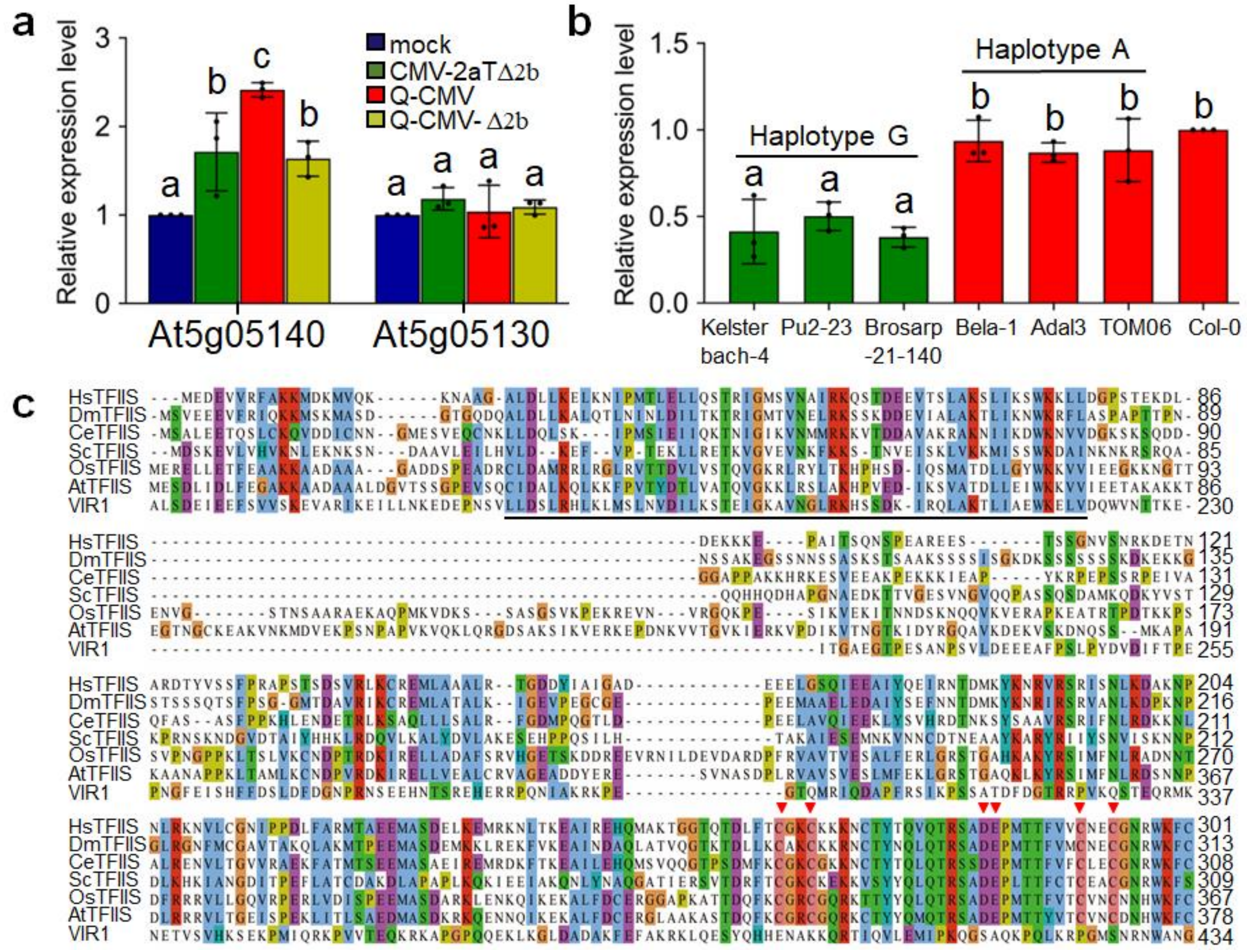

Supplementary Figure 5. VIR1 expression in vivo and protein sequence similarities with eukaryotic TFIISs. a, RT-qPCR analysis of *VIR1* and *AT5G05130* mRNA levels in Col-0 before and after infection with Q-CMV, Q-CMV-Δ2b or CMV-2aTΔ2b. **b**, RT-qPCR analysis of *VIR1* mRNA levels in natural accessions from haplotypes as indicated and Col-0 plants infected with Q-CMV. *VIR1* expression levels were significantly higher in the selected accessions of haplotype A than those from haplotype G after Q-CMV infection. Data in **a** and **b** are means ± SEM from three independent experiments letters indicate significant differences (one-way ANOVA, Duncan, $p<0.05$) and black dots represent the individual values. **c**, *VIR1* sequence similarities with TFIISs from *A. thaliana* (At-NP_181390), rice (Os-ABF99542), yeast (Sc-GAX68285), fruit fly (Dm-NP_476967), nematode (Ce-CAA90943), or human (Hs-NP_006747) aligned by Clustal W. Amino acid positions are given at the right whereas black line and red tringles highlight the conserved domain and essential amino acids in the N- and C-terminal regions, respectively. The source data underlying qRT-PCR in (**a**) and (**b**) are provided as a Source Data file.

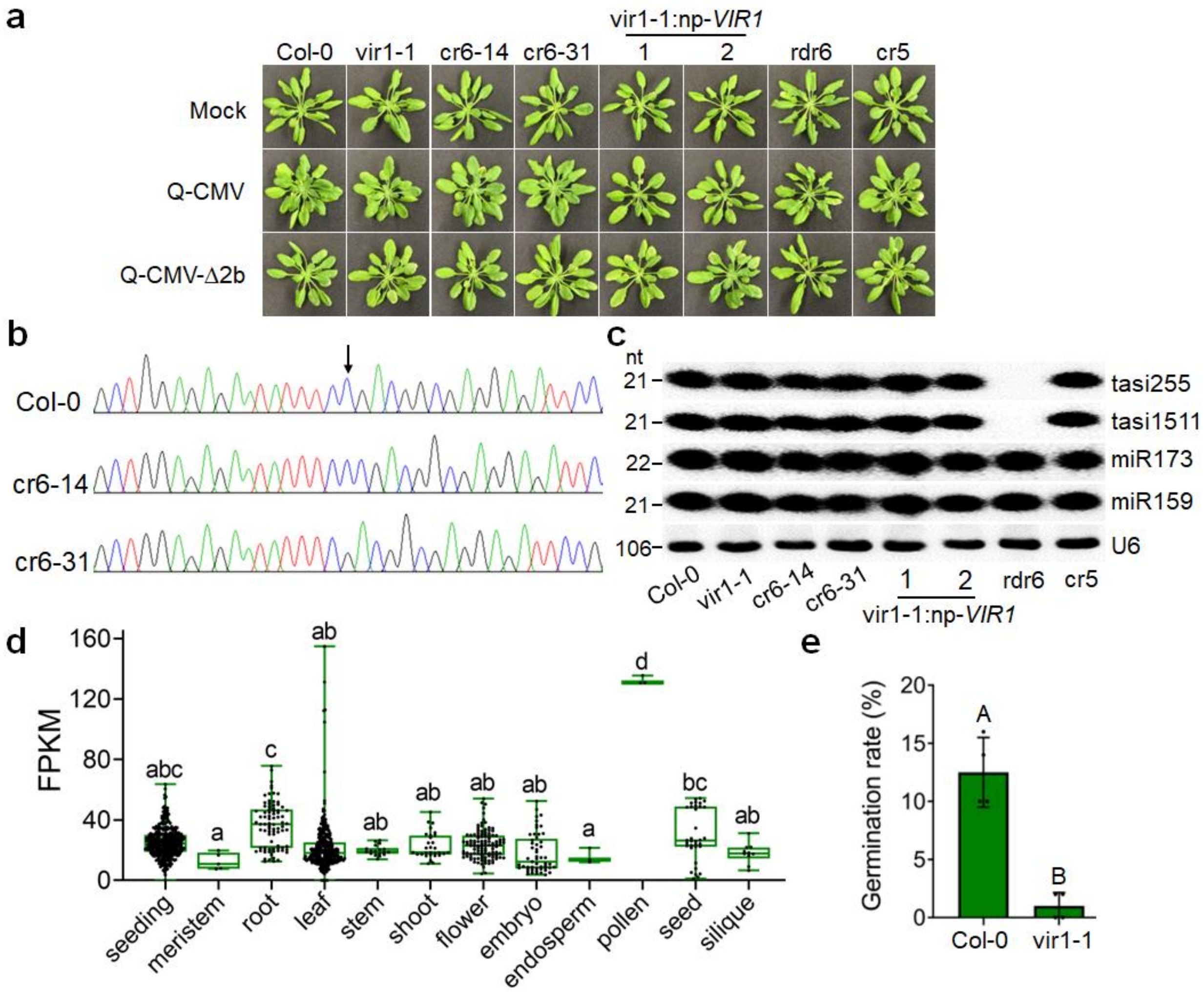

Supplementary Figure 6. Characterization of vir1 mutants. a, Wild-type, *vir1* mutants and *VIR1* complemented plants were photographed 2 weeks post-inoculation with buffer (mock), Q-CMV or Q-CMV-Δ2b. No obvious phenotypic differences were observed among Col-0, *vir1* mutants and the complemented lines with or without infection in contrast to the *rdr6* mutant. **b**, DNA sequencing of Col-0 and the edited mutants *cr6-14* and *cr6-31* containing an insertion and deletion of single base pair (position indicated by a black arrow) in the 15th codon of *VIR1*, respectively. **c**, Northern blot detection of plant endogenous small RNAs (tasi255, tasi1511, miR173 and miR159) in healthy wild-type and mutant plants as well as the complemented lines as indicated, the experiment was repeated three times independently with similar results. **d**, Expression pattern of *VIR1* in different tissues of *A. thaliana* shows in fragments per kilobase of transcript per million mapped reads (FPKM). Boxplot represents 25th percentiles and 75th percentiles with median as center line and whiskers represent the date from minima to maxima, black dots represent all individual experiments and letters indicate significant differences (one-way ANOVA, Duncan, n=960 libraries and $p<0.05$). **e**, Seed dormancy assay to determine the germination rates of Col-0 and *vir1-1*. Data are means ± SEM from four replicates and letters indicate significant differences (two-sided t test, $p=0.0004$). The source data underlying virus infection symptoms in (**a**), blots in (**c**), FPKM value data in (**d**) and germination rates in (**e**) are provided as a Source Data file.

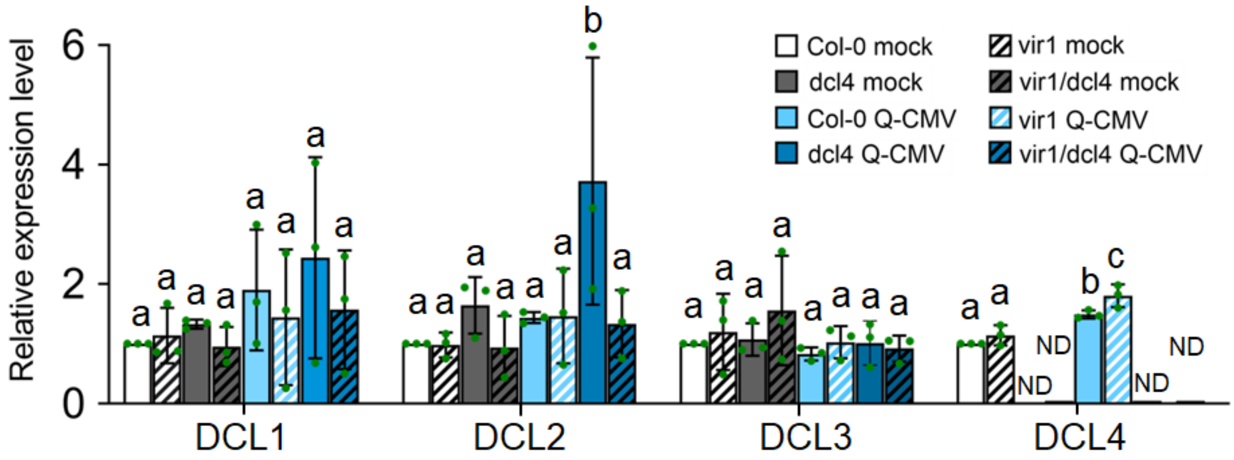

Supplementary Figure 7. DCL1, DCL2, DCL3 and DCL4 expression in vivo. RT-qPCR analysis of the four DCLs mRNA levels in wild-type (Col-0), single or double mutant plants without infection or one week post-infection with Q-CMV as indicated. *A. thaliana* EF1α (At5g60390) mRNA was used as internal control. Data are means ± SEM from three independent experiments letters indicate groups with significant differences (one-way ANOVA, Duncan, p<0.05), ND, not determined and green dots represent the individual values. The source data underlying qRT-PCR data are provided as a Source Data file.
