# Supplementary material for: Identification of positive and negative regulators of antiviral RNA interference in Arabidopsis thaliana
Source: Nat Commun. 2022 May 30;13:2994. doi: 10.1038/s41467-022-30771-0 (PMC9151786; doi:10.1038/s41467-022-30771-0)
Supplement: Supplementary file 3 — Description of Additional Supplementary Files [file 41467_2022_30771_MOESM3_ESM.pdf]

Description of Additional Supplementary Files

File Name: Supplementary Data 1
Description: Accumulation levels of Fny-CMV-Δ2b determined by ELISA (log- transformed) and of Q-CMV by RT-qPCR in A. thaliana accessions with the assigned haplotype of RDO5 or VIR1.

File Name: Supplementary Data 2
Description: Sequence and usage of primer pairs.
