# Supplementary material for: Identification of positive and negative regulators of antiviral RNA interference in Arabidopsis thaliana
Source: Nat Commun. 2022 May 30;13:2994. doi: 10.1038/s41467-022-30771-0 (PMC9151786; doi:10.1038/s41467-022-30771-0)
Supplement: Supplementary file 6 — Reporting Summary [file 41467_2022_30771_MOESM6_ESM.pdf]

nature portfolio

Corresponding author(s): Shou-Wei Ding, Zhenyu Jia

Last updated by author(s): May 12, 2022

Reporting Summary

Nature Portfolio wishes to improve the reproducibility of the work that we publish. This form provides structure for consistency and transparency in reporting. For further information on Nature Portfolio policies, see our Editorial Policies and the Editorial Policy Checklist.

Statistics

For all statistical analyses, confirm that the following items are present in the figure legend, table legend, main text, or Methods section.

n/a	Confirmed	
☐	☒	The exact sample size (*n*) for each experimental group/condition, given as a discrete number and unit of measurement
☐	☒	A statement on whether measurements were taken from distinct samples or whether the same sample was measured repeatedly
☐	☒	The statistical test(s) used AND whether they are one- or two-sided<br>*Only common tests should be described solely by name; describe more complex techniques in the Methods section.*
☒	☐	A description of all covariates tested
☐	☒	A description of any assumptions or corrections, such as tests of normality and adjustment for multiple comparisons
☐	☒	A full description of the statistical parameters including central tendency (e.g. means) or other basic estimates (e.g. regression coefficient) AND variation (e.g. standard deviation) or associated estimates of uncertainty (e.g. confidence intervals)
☐	☒	For null hypothesis testing, the test statistic (e.g. *F*, *t*, *r*) with confidence intervals, effect sizes, degrees of freedom and *P* value noted<br>*Give P values as exact values whenever suitable.*
☒	☐	For Bayesian analysis, information on the choice of priors and Markov chain Monte Carlo settings
☒	☐	For hierarchical and complex designs, identification of the appropriate level for tests and full reporting of outcomes
☐	☒	Estimates of effect sizes (e.g. Cohen's *d*, Pearson's *r*), indicating how they were calculated

Our web collection on statistics for biologists contains articles on many of the points above.

Software and code

Policy information about availability of computer code

Data collection	ELISA data reading: infinite 200 Pro plate reader (Tecan i-control).<br>RT-qPCR detection: CFX96 Real-Time System (Bio-rad).<br>Northern blot signal measuring: ImageQuant TL 7.0 (GE Healthcare).<br>VIR1 expression pattern in Arabidopsis thaliana: Arabidopsis RNA-Seq Database (http://ipf.sustech.edu.cn/pub/athrna/) (doi:10.1016/j.molp.2020.08.001)
Data analysis	GWAS analyses: easyGWAS pipeline (https://easygwas.ethz.ch/) based on the 1001 genomes data, TAIR10 gene annotations and EMMAX algorithms.<br>Manhattan and quantile-quantile plots preparing: R program (version 3.5.1) with the "qqman" package.<br>Statistical analyses: GraphPad Prism 7.<br>Pearson's correlation: GraphPad Prism 7.<br>Heritability estimate: ANOVA approach.<br>AVI4 multiple sequences alignment: Clustal W.

For manuscripts utilizing custom algorithms or software that are central to the research but not yet described in published literature, software must be made available to editors and reviewers. We strongly encourage code deposition in a community repository (e.g. GitHub). See the Nature Portfolio guidelines for submitting code & software for further information.

Data

Policy information about availability of data

All manuscripts must include a data availability statement. This statement should provide the following information, where applicable:

- Accession codes, unique identifiers, or web links for publicly available datasets
- A description of any restrictions on data availability
- For clinical datasets or third party data, please ensure that the statement adheres to our policy

The data support the findings of this study are available within the manuscript and supplementary information. The original figures of blots and raw data underlying graphs presented in the paper are available in source data file.

Field-specific reporting

Please select the one below that is the best fit for your research. If you are not sure, read the appropriate sections before making your selection.

[x] Life sciences [ ] Behavioural & social sciences [ ] Ecological, evolutionary & environmental sciences

For a reference copy of the document with all sections, see nature.com/documents/nr-reporting-summary-flat.pdf

Life sciences study design

All studies must disclose on these points even when the disclosure is negative.

Sample size	16 plants of the same accession or genotype plants were used for virus inoculation, samples collected from those 16 plants were pooled for downstream detection. 496 and 500 Arabidopsis thaliana natural accessions randomly selected from the 1001 Genome Project were used for ELISA and RT-qPCR detection for GWAS analysis, respectively. The amount of natural accessions used in this study are more than double size of accessions used in numerous publications conducting GWAS in Arabidopsis thaliana.
Data exclusions	No data were excluded.
Replication	The number of independent replicates is indicated in the figure legend and all experiments were repeated at least three times.
Randomization	Arabidopsis natural accessions were randomly selected without bias from the 1001 genome program.
Blinding	Due to the nature of experimental setup, our results are objective and not rely on subjective judgment, blinding is not reasonably applicable.

Reporting for specific materials, systems and methods

We require information from authors about some types of materials, experimental systems and methods used in many studies. Here, indicate whether each material, system or method listed is relevant to your study. If you are not sure if a list item applies to your research, read the appropriate section before selecting a response.

Materials & experimental systems

n/a	Involved in the study	
[ ]	[x]	Antibodies
[x]	[ ]	Eukaryotic cell lines
[x]	[ ]	Palaeontology and archaeology
[x]	[ ]	Animals and other organisms
[x]	[ ]	Human research participants
[x]	[ ]	Clinical data
[x]	[ ]	Dual use research of concern

Methods

n/a	Involved in the study	
[x]	[ ]	ChIP-seq
[x]	[ ]	Flow cytometry
[x]	[ ]	MRI-based neuroimaging

Antibodies

Antibodies used	Mouse monoclonal antibody specific to Fny-CMV Coat protein (doi:10.1016/j.jviromet.2004.09.014), was kindly provided by Dr. Xueping Zhou, Zhejiang University. 1:2000 dilution for ELISA and 1:5000 dilution for western blot.<br>Mouse monoclonal antibody specific to tubulin (Agrisera, AS20 4483). 1:5000 dilution for western blot.<br>Goat anti-mouse IgG (H+L) secondary antibody (Invitrogen G-21040). 1:2000 dilution for western blot.<br>Alkaline phosphatase-conjugated Anti-Mouse secondary antibody (Invitrogen, WP20006). 1:2000 dilution for ELISA.
Validation	Mouse monoclonal antibody specific to Fny-CMV CP was used fro Fny-CMV Coat protein detection according to previous study (doi:10.1016/j.jviromet.2004.09.014).<br>Mouse monoclonal antibody specific to tubulin (Agrisera, AS20 4483) was used for Arabidopsis thaliana tubulin detection as loading

control. Manufacturer validated for western blot with Arabidopsis thaliana samples in numerous publications
Goat anti-mouse IgG (H+L) secondary antibody was used for western blot detection of Fny-CMV Coat protein and A. thaliana tubulin.
Alkaline phosphatase-conjugated Anti-Mouse secondary antibody was used for ELISA detection of Fny-CMV Coat protein.
